# Sir Joseph Lister

**Published:** 1894-12

**Authors:** J. Fredk. W. Silk

**Affiliations:** Honorary Secretary


### TESTIMONY TO SIR JOSEPH LISTER.

Sir Joseph Lister, having recently retired from active hospital and teaching work, the occasion has been thought appropriate for presenting him with a testimonial of the esteem in which he is held by his former colleagues and pupils, and committees have, therefore, been formed in Glasgow, Edinburgh and London, for the purpose of raising the necessary funds.

It is proposed that the testimonial will take the form of a portrait. Subscriptions have been limited to two guineas, and it is hoped that sufficient funds will be collected to permit of some memento of the occasion being presented to each subscriber of that amount.

As there are probably many surgeons in the United States who may wish to join in the movement, but whose names and exact addresses it has been difficult to ascertain, I should be glad if you would permit me to state that subscriptions may be sent to me at 29 Weymouth street, Portland Place, London, W. England; or to one or other of the following gentlemen, who have kindly consented to act as treasurers, viz.: Dr. James Finlayson, 4 Woodside Place, Glasgow, Scotland; Professor Chiene, 26 Charlotte Square, Edinburgh, Scotland; Professor William Rose, 17 Harley street, London, W. England; Dr. Malloch, 124 James street, South Hamilton, Ontario; or J. Stewart, M. B., 37 South street, Halifax, Nova Scotia.

I have the honor to remain, sir,

Yours, faithfully,

J. FREDK. W. SILK,

Honorary Secretary.

P.S—Two guineas are about $10.23.

